# Individual *in vivo* Profiles of Microglia Polarization After Stroke, Represented by the Genes iNOS and Ym1

**DOI:** 10.3389/fimmu.2019.01236

**Published:** 2019-06-04

**Authors:** Franziska M. Collmann, Rory Pijnenburg, Somayyeh Hamzei-Taj, Anuka Minassian, Kat Folz-Donahue, Christian Kukat, Markus Aswendt, Mathias Hoehn

**Affiliations:** ^1^In-vivo-NMR, Laboratory, Max Planck Institute for Metabolism Research, Cologne, Germany; ^2^FACS & Imaging Core Facility, Max Planck Institute for Biology of Ageing, Cologne, Germany; ^3^Department of Neurology, University Hospital Cologne, Cologne, Germany; ^4^Radiology Department, Leiden University Medical Center, Leiden, Netherlands; ^5^PERCUROS, Enschede, Netherlands

**Keywords:** microglia, polarization phenotype, M1-like phenotype, M2-like phenotype, iNOS, Ym1, bioluminescence imaging, stroke

## Abstract

Microglia are the brain-innate immune cells which actively surveil their environment and mediate multiple aspects of neuroinflammation, due to their ability to acquire diverse activation states and phenotypes. Simplified, M1-like microglia are defined as pro-inflammatory cells, while the alternative M2-like cells promote neuroprotection. The modulation of microglia polarization is an appealing neurotherapeutic strategy for stroke and other brain lesions, as well as neurodegenerative diseases. However, the activation profile and change of phenotype during experimental stroke is not well understood. With a combined magnetic resonance imaging (MRI) and optical imaging approach and genetic targeting of two key genes of the M1- and M2-like phenotypes, iNOS and Ym1, we were able to monitor *in vivo* the dynamic adaption of the microglia phenotype in response to experimental stroke.

## Introduction

Stroke is still one of the leading causes of death, yet without applicable therapeutics. On a global level, 1 in 6 people are at risk of stroke, while 15 million patients per year suffer from stroke (1). The innate immune system plays an essential role, long neglected, in stroke pathology and there is an urgent need to unravel the underlying mechanisms of action. Microglia, the resident brain immune cells, constantly scan their microenvironment and immediately react to changes in their environment. Their action is highly dynamic and dependent not only on their localization, but also on the stimuli and temporal profile after insult (2, 3).

Microglia and infiltrating monocyte-derived macrophages (MDMs) are activated after stroke (4–8) and highly influence each other and nearby cells through secretion of cytokines that can be both, neuroprotective and cytotoxic (9, 10). The majority of experimental stroke studies agrees on a preceding microglia activation, followed by MDM accumulation (11–13).

Earlier studies suggested that in the very early state after stroke, microglia secrete predominantly toxic factors, such as pro-inflammatory cytokines [e.g., interleukin-1β (IL-1β), tumor necrosis factor-α (TNF-α), interleukin-6 (IL-6)], and reactive oxygen species (ROS), resulting in the (simplified) classification of so-called M1-like phenotype (14). The resulting increase in tissue damage causes further microglia activation, which promotes even more neuronal loss (15). At this stage, systemic immune cells start to infiltrate to support local inflammation. Infiltrating MDMs phagocytose debris and release growth factors and anti-inflammatory cytokines, such as insulin-like growth factor (IGF)-1. This so-called alternatively-activated or M2-like phenotype can suppress and regulate microglial activation (3, 16). Microglia/macrophages acquire an anti-inflammatory M2-like phenotype, which appears to be taken over by the pro-inflammatory phenotype over time (4). Notably, until recently, due to missing markers in *post-mortem* studies, microglia were barely distinguishable from MDMs, leading to variable findings of activation stages in these two unique cell types in the context of the temporal profile suggesting that the M1-like and M2-like phenotypes are rather extremes of mixed phenotypes (14). Furthermore, using flow cytometry, Wattananit et al. reported on an increased anti-inflammatory response of MDMs at 14 days post stroke (dps), while the pro-inflammatory response in microglia increased from 3 to 7 dps (7).

Several *in vivo* PET or SPECT studies have focused on microglia activation by using translocator protein (TSPO) ligand tracers (17). But until now, studies in the living brain to discriminate between the pro- and anti-inflammatory polarization dynamics in real-time after stroke have not been performed. Moreover, several *ex vivo* studies have reported on pro- and anti-inflammatory markers without taking cell specificity into account. Under ischemic stroke or traumatic brain injury, two recent reports found an early increased anti-inflammatory microglia/macrophage phenotype with a peak at 5 dps, which then gradually switched toward a pro-inflammatory state (4, 18); for reviews refer to (19, 20).

In our present study, we investigated induced nitric oxide synthase (iNOS) and chitinase-like protein-1 (Ym1) activity, representing the M1-like and the M2-like state, respectively, under *in vivo* conditions. With our approach of lentiviral injection prior to stroke induction, we monitor exclusively brain resident cells exclusively, without signal contribution from MDMs.

Here, we show for the first time the temporal *in vivo* profile of increased iNOS and Ym1 promoter activity in the murine brain after stroke by bioluminescence imaging (BLI). Using magnetic resonance imaging (MRI) in parallel, we correlated our findings to the respective lesion volumes.

## Materials and Methods

### Animals

All animal experiments were approved by the local authorities [Landesamt für Natur, Umwelt und Verbraucherschutz North Rhine Westphalia (LANUV)] and conducted according to the German Animal Welfare Act under the animal permission: 84-02.04.2014.A226. All experiments were conducted with male 8–10 week old Rj:NMRI-Foxn1^nu/nu^ mice obtained from Janvier Labs, Le Genest-Saint-Isle, France. Mice were kept in a 12/12 h light/dark cycle and had access to water and food *ad libitum*. During anesthesia, eyes were protected from drying out and mice were kept warm on a heating plate. During MRI scans and surgery the body temperature was monitored via a rectal probe.

### Experimental Protocol

**Figure 1:**
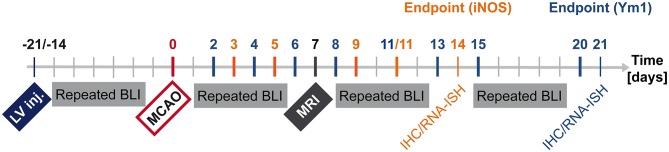
Timeline summarizing *in vivo* and *ex vivo* investigations after LV-iNOS/Ym1-IR injections. BLI signal intensities were repeatedly recorded before stroke induction (MCAO), and at highlighted time points of the respective study group after MCAO (LV-iNOS-IR mice in red, LV-Ym1-IR mice in blue). MRI was conducted 1 week post stroke. Brains were prepared for IHC and RNA-ISH at 14 dps (LV-iNOS-IR mice) or at 21 dps (LV-Ym1-IR mice). BLI, Bioluminescence Imaging; dps, days post stroke; IR, imaging reporter (Luc2-T2A-eGFP); RNA-ISH, RNA *in situ* hybridization.

The lentivirus designed to co-express bioluminescence reporter Luc2 and fluorescence reporter eGFP (abbreviated IR for imaging reporters) driven by the pro- or anti-inflammatory promoters was stereotactically injected into mouse brain striatum. During the following 2–3 weeks, stable integration of the virus was monitored with bioluminescence imaging (BLI). Then, stroke was induced. One week later, MRI was recorded to verify successful lesion induction. BLI was performed repeatedly from the time of stroke induction until 14 days post stroke for the pro-inflammatory reporter, iNOS, and until 20 days post stroke for the anti-inflammatory reporter, Ym1. Animal brains were processed for immunohistochemistry and RNA *in situ* hybridization (Figure 1).

### Anesthesia and Analgesia

Mice that underwent intracranial injections of lentiviral (LV) particles were anesthetized with a combination of Ketamine (85 mg/kg; 100 mg/mL, Ketavet, Pfizer, New York City, NY, USA), Xylazine (18 mg/kg; 2% w/v, Rompun, Bayer Vital GmbH, Leverkusen, Germany), and Acepromazine (3 mg/kg; 1% Vetranquil, Ceva, Tiergesundheit GmbH, Duesseldorf, Germany) in 0.9% Sodium chloride (NaCl) solution (B. Braun Melsungen AG, Melsungen, Germany). Stroke induction and perfusion for brain fixation were conducted under Isoflurane.

Mice were anesthetized in an Isoflurane-flooded knock-out box (3–3.5% for MCAO/BLI/MRI, 4% for perfusion, 7/3 ratio of N_2_O/O_2_) first, and stroke surgery was conducted at 1.5%, brain perfusion at 4% Isoflurane. Prior to surgery, anesthetized mice received analgesia by subcutaneous (s.c.) injection of Carprofen (4 mg/kg (diluted 1:100 in NaCl, 0.9%); 50 mg/mL, Rimadyl, Zoetis, Parsippany-Troy Hills Township, NJ, USA). After surgery, analgesia was provided by Tramal (1 mg/mL diluted in drinking water; 100 mg/mL, Grünenthal GmbH, Aachen, Germany) given for 3 days, freshly prepared every day.

At the end of surgery, mice were subcutaneously (s.c.) injected with 0.9% NaCl solution (10 μL/g body weight) and recovered in a warming cabinet. Health status was checked twice a day during the first 3 days, and later on a regular basis. Wet food was provided for at least the first 2 days after surgery. If necessary, mice received s.c. injections of NaCl, 0.9%.

### Intracranial LV Injections

Concentrated LV particles were produced as described earlier (21), and injected with glass capillaries (cat. no. B100-75-10; Science Products GmbH, Hofheim, Germany), which were pulled on a P-1000 micropipette puller (Sutter Instrument, Novato, CA, USA) and blunt-end cut with a glass knife. Tip quality, length and diameter of each capillary were inspected under the microscope and measured before *in vivo* injections, with tip diameters ranging between 25 and 85 μm. Capillaries were prepared for surgery according to the protocol by Inquimbert et al. (22).

Anesthetized mice were fixed in a stereotactic frame located in a Class 2 Biosafety cabinet. After a small incision of the region slightly above the ears, the skin on top of the skull was gently removed. From the position of the bregma, the striatal target region (anterior-posterior (AP) +0.5 mm; medial-lateral (ML) −2 mm, dorsal-ventral (DV) −3.2/−3 mm) was determined (Digital Just for Mouse Stereotaxic Instrument, Stoelting) and a hole drilled. The dura mater was gently removed, and the glass capillary was inserted 3.2 mm deep from the top of the brain, and immediately pulled up to 3 mm depth. Two microliter of LV particle suspension were injected with a speed of 200 nL/min (min) using a digital stereotaxic injector (QSI, Stoelting Europe). The capillary was left in place for 5 min after injection and then smoothly pulled back up over a time interval of about 4 min. The skin was sutured using an undyed and absorbant 5/0 gauge suture (PGA resoquick, RESORBA Medical GmbH, Nuremberg, Germany), suitable for BLI during the early phase after surgery.

### Stroke Induction

Ischemic stroke was induced with the intraluminal filament model as described earlier (23). In short, the right common carotid artery (CCA), the external carotid artery (ECA), and the internal carotid artery (ICA) were exposed. A silicone rubber-coated filament with a length of 20 mm and a tip diameter of 170 μm (cat. no. 7017PK5Re, Doccol Corporation, Sharon, MA, USA) was introduced through a small incision in the CCA and inserted further into the ICA up to 9 mm distal to the CCA bifurcation to occlude the entrance to the middle cerebral artery (MCA). The incision was sutured, and the animals were allowed to wake up in a 37°C recovery box. After 30 min occlusion time, mice were re-anesthetized with Isoflurane and the filament was withdrawn. The incision in the CCA was sealed by electrocoagulation.

To validate successful stroke induction, T2-weighted MRI was performed at the first week post reperfusion, thus reliably demonstrating the successful MCA occlusion and demarcating the ischemic territory in 3D extent. This assessment protocol is preferable to relying on Laser Doppler Flow measurements which only probe only cortical flow changes in one rather small particular spot without providing information on extent of ischemic territory. The MRI protocol on the other hand had extensively proven reliable in earlier studies in our hands for validating successful MCA occlusion and lesion extent (23–25), where earlier validations with histology and metabolic imaging also demonstrated that MRI is the ideal method to validate successful occlusion and lesion territory.

### Bioluminescence Imaging

Mice were intraperitoneally (i.p.) injected with 300 mg/kg of D-luciferin (Aswendt et al., 2013) (cat. no. bc218, sodium salt 96%, stock 50 mg/mL dissolved in DPBS– and sterile filtered through a 0.2 μm cellulose acetate filter) using an insulin syringe, followed by anesthesia in an Isoflurane-flooded box. 3 min post injection animals were recorded for 30 min in the IVIS SpectrumCT *in vivo* imaging system (PerkinElmer, Waltham, MA) with a constant Isoflurane flow of 2% (or with a lower Isoflurane concentration of 1–1.5% due to health status of some stroke-induced mice). Images were analyzed with Living Image software v4.3.1 (PerkinElmer, Waltham, MA, USA) with an auto-exposure time of minimum (min) 0.5 s (s) and maximum (max) 120 s over 15 frames; binning: min. 2, max 8; F/Stop: min. 1, max 8; field of view (FOV): stage in position C. Reliable signal detection was ensured by an acquisition of at least 3,000 counts.

### Analysis of Bioluminescence Imaging

Identical ROIs including all emitted photons were generated and positioned above the heads. For each time point, the maximum value of photon (p) emission, measured as total flux per second (p/s), was analyzed. To determine the background threshold in bioluminescence imaging (BLI), the total flux was extracted from ROIs on the back of mice. For the most unbiased and conservative analysis, animals with high (*n* = 5) and low signals (*n* = 5) from different studies were chosen. The background signal was calculated as the mean of the averaged total flux of 15 frames plus 3-fold the standard deviation of each measurement, resulting in 3.3 × 10^5^ p/s. In LV-iNOS-IR injected mice, BLI signal change after MCA occlusion (MCAO) was quantified by dividing the max total flux of each time point after MCAO by the averaged max total flux measured at the last three time points (14, 17, 20 days post injection/dpi) before MCAO for each animal. In LV-Ym1-IR injected mice, the signal remained below the threshold before MCAO. Here, signals after MCAO were normalized to the maximal signal after MCAO in each individual. Photographs in the figures were overlaid with images of maximal signal during kinetic measurements. For better visualization and comparison of images, presentation look-up tables (LUTs) of photon emission were set to the same scale within the same group of animals.

### Magnetic Resonance Imaging

Magnetic resonance imaging (MRI) was performed at 7 dps on a Biospec 11.7 Tesla animal MRI system (Bruker BioSpin MRI GmbH, Ettlingen, Germany). Radio frequency (RF) transmission was performed with a 500 MHz mouse resonator (outer/inner diameter of 89/72 mm, Bruker BioSpin MRI GmbH) and signal was received through a 500 MHz proton quadrature mouse head surface coil (Bruker BioSpin MRI GmbH).

The mouse head was fixed with a tooth bar and ear bars, and the animal lay in prone position on a cushion controlling the breathing rate. Mice were kept under Isoflurane (1.0–1.5%) during the scan through an inhalation mask. The animal's body temperature was measured via a fiber optic rectal probe (SA Instruments, NY, USA) and kept constant at 37°C ± 1.0°C by an adjustable water circulating system (medres, Cologne, Germany). Breathing rate and body temperature were continuously monitored (1025T System, SA Instruments, NY, USA) and recorded (DASYlab Software, Measurement Computing, Norton, USA). MRI scans were run using the software Paravision (Version 6, Bruker BioSpin MRI GmbH). Tripilot gradient-echo scans (echo time = 2.3 ms; repetition time = 120 ms) ensured proper positioning of the head.

T2-weighted images were acquired with the Turbo (Rapid Acquisition with Refocused Echoes) (RARE) sequence (TR/TE = 5,500 ms/10 ms, 48 slices, 0.3 mm slice thickness, 17.5 × 17.5 mm^2^ FOV, 256 × 256 matrix, 68 × 68 μm^2^ resolution, 40 kHz bandwidth) and with a Multi-Slice Multi-Echo (MSME) sequence (TR/TE = 2,862 ms/10 ms, 16 slices, 0.6 mm slice thickness, 0.3 mm slice gap, 17.5 × 17.5 mm^2^ FOV, 256 × 256 matrix, 68 × 68 μm^2^ resolution, 89 kHz bandwidth).

Acquired images of the MSME scans were converted into quantitative T2 maps using in-house tools written in IDL software [Version 6.4, ITT Visual Information Solutions (now Harris Geospatial Solutions, Berkshire, UK)] by fitting intensities of each voxel to a mono-exponential decay curve.

In order to create stroke lesion incidence maps (overlaid T2 maps for all mice), one transformation matrix was created by co-registering the first echo of the MSME with the anatomical image obtained by the TurboRARE protocol. In a second matrix, the anatomical data were co-registered to an in-house created template of 8 weeks old nude mice (*n* = 21). The two matrices were combined and applied to the T2 maps, resulting in linearly transformed T2 maps. For reliable discrimination of the ischemic lesion, a T2 threshold above normal was determined and voxels with high T2 values (e.g., ventricles) excluded. For this purpose, masks were manually drawn on the co-registered MRI template with ImageJ software to delineate the ventricles and the cortex of the intact hemisphere. These masks were applied to all mice in order to exclude the ventricles from the incidence maps and to calculate the average healthy T2 value. The threshold was set at T2 values elevated by 10% or more above this individual average value. From the co-registered T2 maps and the individual ischemic T2 threshold, stroke incidence maps were created using in-house ImageJ plugins. The stroke volume was calculated from the individual stroke masks with reference to the total brain volume from the nude mouse template.

### Perfusion Fixation

Mice were deeply anesthetized, the thorax was opened, and a needle connected to a drain tube (Venofix 23 gauge, B. Braun) was inserted into the left ventricle of the heart. After cutting the right atrium, mice were perfused with 20 mL of 2X PBS, followed by 20 mL of 4% PFA with a speed of 9 mL/min, using a Genie Kent syringe pump (Kent Scientific Corporation, Torrington, CT, USA).

### Microscopy

Samples were inspected under an epifluorescence microscope (BZ9000 (Biorevo), Keyence, Osaka, Japan) with the software BZ-II Viewer (Version 2.1, Keyence Corporation). Images were further processed in BZ-II Analyzer software (Version 2.2).

### Immunohistochemistry (IHC)

Brains were prepared for IHC as described in (26) and stained following the protocol as described in (27). Sections stained with α-Iba-1 were pre-treated for 20 min with −20°C cold Acetone (Merck Millipore). For mouse α-GFP antigen retrieval was performed thereafter in tri-sodium citrate dihydrate buffer (cat. no. 1.06448.0500, Merck Millipore, 10 mM, pH 6.0). Sections were blocked for 1 h at room temperature (RT) in 5% normal donkey serum (NDS, Jackson ImmunoResearch Laboratories, Inc., West Grove, PA, USA) in 0.25% Triton X-100 (Carl Roth GmbH & Co. KG) in PBS. Primary and corresponding secondary antibodies were diluted in blocking solution (refer to antibody list in Tables 1, 2). Incubation with only the secondary antibody solution served as control.

**Table 1:** Immunohistochemistry (IHC) primary antibody list.

	**Supplier**	**Cat. no**.		**Conc**.
rb α-Iba-1	Wako Chemicals GmbH	019-19741	Polycl.	1:500
mo α-GFAP	Sigma-Aldrich, St. Louis, MO, USA	G3893	Monocl.	1:200
rb α-GFP	Thermo Fisher Scientific, Waltham, MA, USA	A6455	Polycl.	1:200
mo α-GFP	Santa Cruz Biotechnology, Dallas, TX, USA	sc-9996	Monocl.	1:100

*mo, mouse; rb, rabbit*.

**Table 2:** IHC secondary antibody list.

	**Supplier**	**Cat. no**.		**Conc**.
Cy5 do α-mo (GFAP)	JIR Lab., Inc.	715-175-151	Polycl.	1:200
Cy3 do α-rb (Iba-1)	JIR Lab., Inc.	711-165-152	Polycl.	1:200
FITC go α-mo (GFP)	JIR Lab., Inc.	115-096-068	Polycl.	1:100
AlexaFluor 488 do α-rb (GFP)	Thermo Fisher Scientific	A-21206	Polycl.	1:200

*AffiniPure IgG (H + L) antibodies. do, donkey; go, goat; mo, mouse; rb, rabbit*.

### RNA *in situ* Hybridization (RNA-ISH)

Fixed and frozen brain sections were processed using the RNAScope Multiplex Fluorescent v2 kit (cat. no. 323110, Advanced Cell Diagnostics (ACD), Newark, CA, USA) following the user's manuscript with a few modifications. In short, slides were washed in DPBS–, followed by diethyl pyrocarbonate (DEPC)-H_2_O. Endogenous peroxidases were quenched by H_2_O_2_ (cat. no. 322281, ACD) for 10 min at RT followed by washing in dH_2_O. Epitopes were retrieved by cooking in Target retrieval reagent (cat. no. 322000, ACD) for 5 min at 98–100°C. Slides were briefly rinsed in dH_2_O, followed by 100% ethanol (EtOH). After drying at RT, slides were incubated with Protease III (cat. no. 322281, ACD) for 40 min at stable 40°C in the HybEZ Hybridization system oven (ACD), followed by washing in dH_2_O. Probes (Probe diluent, i.e., control probe (cat. no. 300041) in C1 channel, Luc2 in C2 channel, Iba-1 in C3 channel) were hybridized for 2 h at 40°C. RNA-ISH 3-plex positive (cat. no. 320881, ACD) and negative control probes (cat. no. 320871, ACD) were included in all experiments to assess signal-to-noise ratio. Slides were stored in 5X sterile saline-sodium citrate (SSC) buffer (see Table 3) over night (ON) at RT, and the next day, pre-amplification AMP1 to AMP3 probes were successively hybridized. Signals were consecutively developed and amplified with HRP-conjugated antibodies directed against the pre-amplification probes, and further amplified by Tyramide signal amplification (TSA) fluorescent probes (cat. no. NEL741001KT, Perkin Elmer), diluted in Multiplex TSA buffer (cat. no. 322809, ACD) (see dilution in Table 3). After TSA incubation horse radish peroxidase (HRP) availability was blocked by HRP blocker (ACD). Slides were washed again in 1X wash buffer and then incubated in DAPI (ACD) for 30 s. Brains were mounted in ProLong gold antifade mountant with DAPI (Thermo Fisher Scientific) and stored ON in the dark. Hybridized brain sections were inspected under the microscope, and for each channel, exposure time was assessed in negative control sections first.

**Table 3:** RNAScope probe list.

	**Supplier**	**Conc**.
Probe diluent	Advanced Cell Diagnostics (ACD), Newark, CA, USA	
C2 (Luc2)	ACD	1:50
C3 (Iba-1)	ACD	1:50
3-plex pos. ctrl. containing:	ACD	1 drop
POLR2A (C1, low)		
PPIB (C2, middle)		
UBC (C3,high)		
**TSA plus fluorophores**		
TSA buffer in C1	PerkinElmer, Waltham, MA, USA	
Cyanine Cy3 in C2	PerkinElmer	1:1,500
Cyanine Cy5 in C3	PerkinElmer	1:750
**SSC buffer (20X)**		
NaCl	175.3 g	
Na_3_C_6_H_5_O_7_	88.2 g	
dH_2_O	ad 800 mL	
Adjust pH to 7.0 with HCl, 1 M		
dH_2_O	ad 1,000 mL	

*C, channel; ctrl, control*.

### Flow Cytometry of Isolated Brain Cells

Cells of individual mice were isolated using the Adult Brain Dissociation kit (cat. no. 130-107-677, Miltenyi Biotec GmbH, Bergisch Gladbach, Germany). After cervical dislocation brains were placed into cold DPBS including calcium, magnesium, glucose, and pyruvate (cat. no. 14287080, Thermo Fisher Scientific). A small region of 2 mm coronal thickness including the injection area was isolated. Brain tissue injected with HBSS only served as control for autofluorescence. Samples were acquired using a BD LSRFortessa analyser (BD Biosciences) with the software BD FACSDiva (Version 8.0.1., BD Biosciences).

Tissue samples were weighed and further processed for cell isolation following the users manual with a few modifications due to the small size of the samples. In short, brain tissue was homogenized in the provided enzymatic mix for 30 min at 37°C, followed by filtering dissociated samples through a 70 μm strainer. A density gradient was applied and the top two phases were carefully aspirated. After washing in DPBS, red blood cells were removed for 10 min at 4°C. Five milliliter of PB staining buffer (0.5% bovine serum albumin (BSA) in MACS Rinsing solution) were added, and after centrifugation at 300 × g for 10 min at 4°C, supernatant was discarded and the cell pellet re-suspended in PB staining buffer. Cells of each individual mouse brain were counted on an automated cell counter. The cell solution was split and a negative control probe, stained with Hoechst 33342 staining (1:100, cat. no. 130-111-569, Miltenyi Biotec GmbH) and propidium iodide (PI) solution (1:100, cat. no. 130-093-233, Miltenyi Biotec GmbH) only, served as background control. Cells were incubated with CD11b-PE-Vio770 (1:11, cat. no. 130-109-365, Miltenyi Biotec GmbH) and CD45-APC (1:50, cat. no. 130-110-798, Miltenyi Biotec GmbH) for 10 min at 4°C. After washing in PB buffer and centrifugation, the cell pellet was re-suspended in PB buffer and incubated with Hoechst for 15 min at RT. Shortly before analysis, PB buffer was added to a final volume of 300 μL, and PI added. Cells were filtered through a 50 μm filter and were immediately analyzed. For proper compensation settings to correct for fluorescence spillover, MACS Comp Beads (MACS Comp Bead kit, anti-REA, Miltenyi Biotec GmbH) were applied to each fluorochrome-conjugated antibody solution. MACS Comp Beads—blank served as the negative control. Beads were incubated with antibodies in autoMACS Running buffer (Miltenyi Biotec GmbH) for 5–10 min at RT. Bead samples were diluted with 1 mL of autoMACS Running buffer and processed for compensation adjustments. Cell populations were analyzed with FlowJo software (Version 10.0.7, FlowJo LLC, Ashland, OR, USA). Single cells were gated based on forward scatter height (FSC-H) and FSC-area (A) and from here, mononuclear cells (MNCs) were identified by side scatter (SSC)-A and FSC-A. To exclude dead cells, mononuclear cells were further separated based on PI and FSC-A.

### Statistics

Raw BLI data were analyzed in the IBM SPSS Statistics software (Version 22, IBM, Armonk, NY, USA) and tested for normal distribution with the Shapiro Wilk test and a significance level of α = 0.05. Data without normal distribution were tested for a general significant effect by the nonparametric Friedman test in SPSS or—when data sets were missing—by the Skillings-Mack test in the XLSTAT software (free version at https://www.xlstat.com/de/), followed by the *post hoc* Wilcoxon Signed-Rank test. The confidence interval was set to 99%.

Correlation tests were performed with the Graphpad Prism Software (Version 7.03, GraphPad Software, La Jolla, CA, USA). The coefficient of determination, R^2^, based on the Pearson correlation coefficient r, was computed with two-tailed *p*-values with a confidence interval of 95%.

## Results

### Assessment of Stable Reporter Expression by Virus Integration

**Figure 2:**
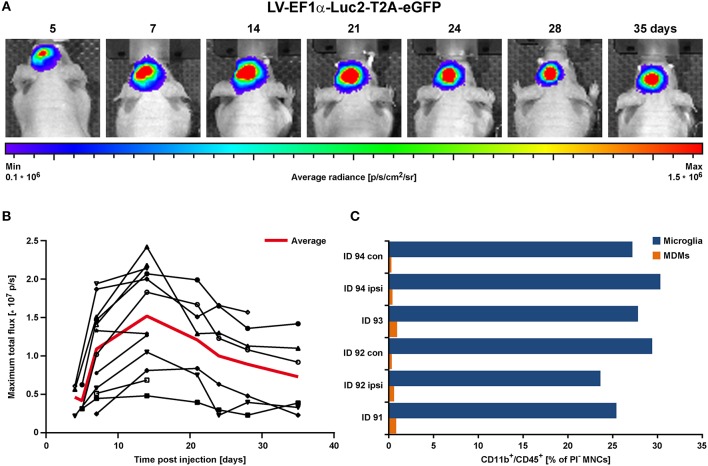
BLI proof of concept in the healthy brain *in vivo*. LV-EF1α-IR particles were injected into the striatum to assess the timing for stable Luc2 expression. **(A)** Representative images of BLI signal behavior over time in one individual. Shown is the frame with maximal signal, overlaid with the corresponding photographs. **(B)** Quantitative BLI analysis of maximum total flux in photons per second (p/s). Signal reached steady state after about 14 days and remained stable until at least 35 dpi. The red line depicts the average values of all animals, and error bars indicate STDs. **(C)** Flow cytometry analysis of cells isolated at 2 weeks post LV injection. Numbers of both, microglia and MDMs remained independent of treatment. Irrespective of injected solutions [HBSS (ID 94) vs. LV] or insertion of a capillary [injection vs. no injection (con)], microglia numbers were similar and MDMs were less present. con, contralateral; dpi, days post injection; ipsi, ipsilateral; MDMs, monocyte-derived macrophages; STD, standard deviation.

Concentrated LV-EF1α-IR particles were injected into the striatum of 8–9.5 weeks old healthy nude mice (*n* = 12) (22). This experiment served to determine the necessary incubation period to reach stable imaging reporter expression. Bioluminescence imaging (BLI) measurements were repeatedly conducted over a 5 week period. A rapid BLI signal increase was detected in the first 2 weeks, followed by a slow decrease thereafter (Figures 2A,B).

In additional animals (*n* = 3), cells were isolated from 2 mm thick coronal brain sections of the injected hemispheres at 15 days post LV injection and prepared for flow cytometry of individual brain sections. With this, we assessed the contribution of CD11b^+^/CD45^high^ infiltrating monocyte-derived macrophages (MDMs), which are present in response to capillary insertion and LV infection, compared to the presence of CD11b^+^/CD45^low^ brain resident microglia (gating strategy in Supplementary Figure S1). One hemisphere injected with HBSS only (ID 94) and the untreated contralateral hemispheres served as control. In addition, the majority of immune cells detected by flow cytometry were microglia with frequencies between 23.6–30.3% compared to MDMs with frequencies below 1% (Figure 2C).

In summary, LV injection did not initiate an additional immune response (e.g., proliferation of resident microglia or infiltration of MDMs) at 2 weeks post injection. These observations indicate an only weak immune response by infiltrating MDMs on 8 dpi (HBSS injected control brain) or 15 dpi. This weak inflammatory response may be attributed to the use of very thin capillaries producing only very little damage, or to the disappearance of MDMs already by the time of analysis 2 weeks after LV injection. Thus, only a negligible number of transduced MDMs may contribute to the later microglia BLI signal after stroke induction. Furthermore, the stable BLI signals over 5 weeks post LV injection assured a stable LV integration over an extended time window, clearly long enough to study Luc2 cell activity for at least 3 weeks post stroke onset.

### BLI *in vivo* Imaging After Stroke

**Figure 3:**
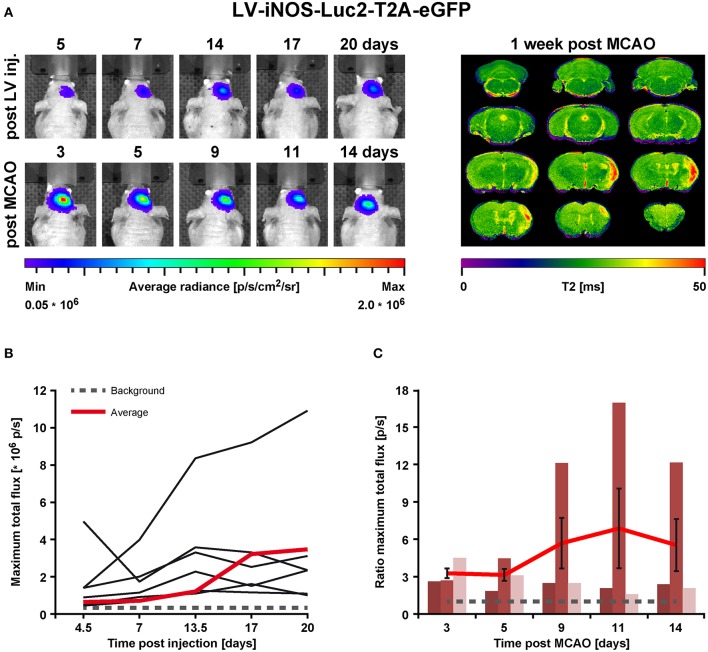
BLI *in vivo* analysis of iNOS activity in LV-iNOS-IR injected brains before and after MCAO. **(A)** BLI signal distribution over time in one representative animal before (upper row) and after MCAO (bottom row) with the corresponding MRI T2 map (right). **(B)** BLI signals, indicated as maximum total flux in p/s, increased during the first 2 weeks after LV injections and, except for one outlier, reached steady state thereafter. The gray dotted line indicates the background threshold. **(C)** After MCAO, BLI signals strongly increased in animals with cortico-striatal lesions with inter-individual variability. For each individual animal, the pre-stroke values at the last 3 days before stroke induction (i.e., at days 14, 17, and 20 post virus injection) were averaged. This average values was used to normalize the post-stroke signal intensities to. For better visualization, the dotted line indicates a ratio of 1. Error bars represent + SEM (*n* = 6 before MCAO) or ± SEM (*n* = 3 after MCAO). IR, imaging reporter (Luc2-T2A-eGFP); p/s, photons per second.

In order to assess the pro- and anti-inflammatory microglia activation state after stroke, concentrated LV particles with the expression constructs iNOS-IR or Ym1-IR, respectively, were intrastriatally injected into 8.5–10 week old nude mice and followed by BLI. Based on the observation of steady signal in the LV-EF1α-IR study after 14 dpi, stroke was induced at 14 or 21 days after LV injection for the LV-Ym1-IR injected mice, or at 21 days after LV injection for LV-iNOS-IR injected mice. Luc2 expression was monitored repeatedly before and after stroke induction at defined time points (Figure 1). BLI measurements before stroke induction assured steady state (LV-iNOS study, Figure 3B) or persistent lack of signals (LV-Ym1 study, Figure 6C). Elevated T2 relaxation times on quantitative MRI maps at 7 days post stroke (dps) demonstrated successful ischemic lesion induction. LV-iNOS-IR and LV-Ym1-IR injected brains were prepared for IHC and RNA-ISH at 14 and 21 dps, respectively, to characterize transduced cells and their location in respect to the lesion area.

### iNOS Up-Regulation After Stroke

In the LV-iNOS-IR injected animals (*n* = 6), BL signals were present as early as 4 or 5 dpi with a slight increase until 14 dpi, in line with BLI observations after LV-EF1α-IR injections. Apart from one animal with BL signal shift toward the olfactory bulb and continuous signal increase, BL signals reached steady state after 14 dpi (Figures 3A,B). BLI signal data over time were tested for significance by the nonparametric Friedman test followed by the Wilcoxon Signed-Rank test. Despite some inter- and intra-individual variations, individual signal intensities were not significantly different between days 14, 17, and 20, i.e., before stroke.

To quantify BLI signal changes after MCAO, the BLI signal intensities at all time points post stroke induction were normalized to the averaged BLI signal intensities of (14, 17, and 20) dpi for each individual (Figure 3C). Based on MRI T2 maps at 7 dps, three of the surviving mice displayed T2 lesion volumes >5% of total brain (/TB), depicting cortico-striatal lesions, and two showed T2 lesion volumes <5%/TB with only small striatal or no visible lesion formation (Supplementary Figure S2, left). Notably, in all animals with cortico-striatal lesions, BLI signals strongly increased (Figure 3) with apparent changes already in the first measurement at 3 dps.

**Figure 4:**
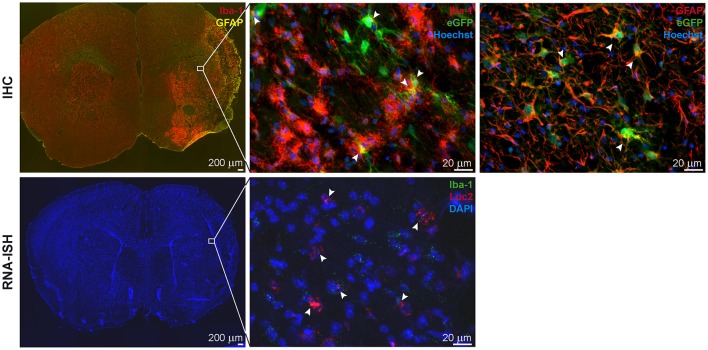
IHC and RNA-ISH of LV-iNOS-IR injected brains at 14 days after MCAO. IHC on top, RNA-ISH below. Iba-1^+^ and GFAP^+^ cells accumulated in the ischemic area **(Left)**. eGFP^+^ cells co-labeled with Iba-1 **(Center)** and GFAP **(Right)** (arrow heads). RNA-ISH confirmed reporter expression: Luc2 RNA positive cells were found in the ischemic hemisphere, upon which some were also positive for Iba-1 RNA (arrow heads). 4X magnifications left; 60X remaining images. IHC, immunohistochemistry; RNA-ISH, RNA *in situ* hybridization.

BLI signal increases were diverse in intensity, and maximal signals were reached at different time points (Figure 3C). In detail, two animals showed maximal BLI signals in the acute state at 3 dps, while in one animal signal increased strongly between 5 and 9 dps and peaked at 11 dps with a 17-fold increase of the pre-stroke signal intensity. In animals without cortico-striatal lesions (n = 2) determined by MRI at 7 dps, BLI signals did not change over time (Supplementary Figure S3). Correlation tests for BLI quantification and T2 lesion volumes measured at 1 week post stroke revealed only a weak, positive correlation of BLI signal integration over the whole observation time (area under curve), indicated as total immune response (*R*^2^ = 0.48; *p* = 0.198). A weak correlation with lesion volume was also found for maximum values of photon emission, ranging from 2.6-fold to 17-fold, indicated as maximal BLI signals (*R*^2^ = 0.43; *p* = 0.232). The time from stroke induction to maximal BLI response showed a clear inverse correlation with lesion volumes (R^2^ = 0.59, p = 0.128) (Figure 4).

In summary, the iNOS promoter is activated by stroke injury and the strength of up-regulation is related to the lesion size. Furthermore, larger lesion size causes an earlier up-regulation of the iNOS activity.

**Figure 5:**
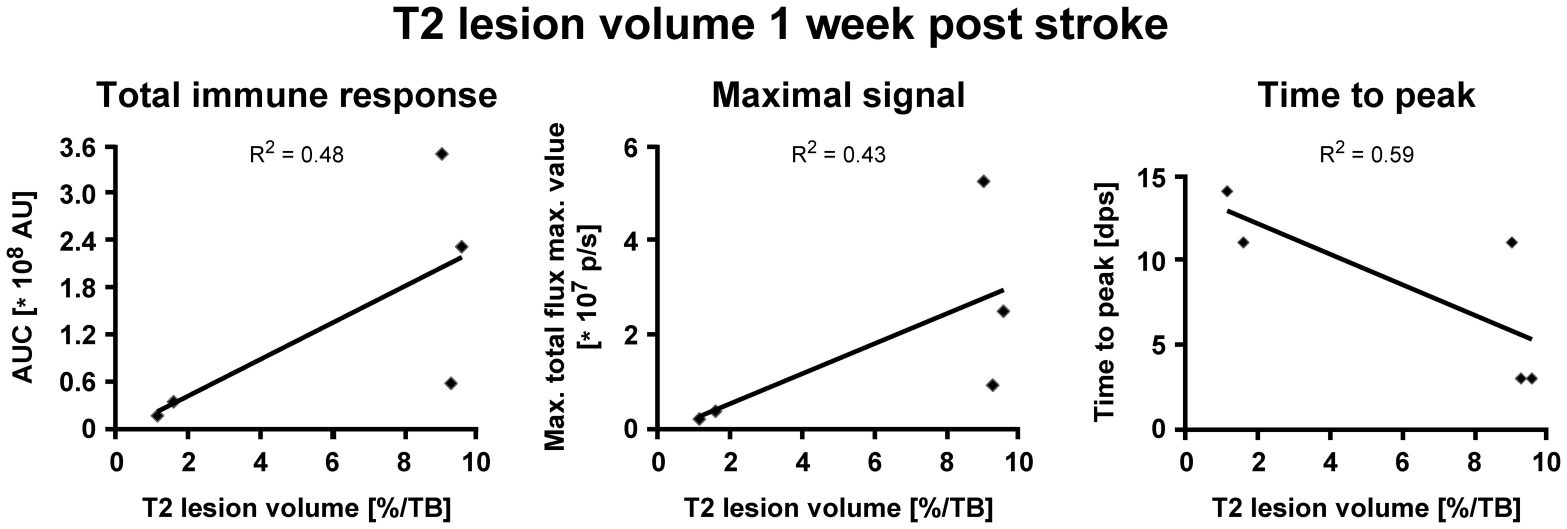
Correlation of iNOS-dependent BL signal intensities and T2 lesion volumes after stroke. T2 lesion volumes were calculated at 1 week post stroke. **(A)** BLI signal integration over time, designated as area under curve (AUC), and **(B)** the maximum values of emitted photons moderately positively correlated with T2 lesion volumes. **(C)** The time to peak negatively correlated with T2 lesion volumes. AU, arbitrary units; dps, days post stroke.

In all animals with cortico-striatal lesions, eGFP^+^ cells were detected by IHC in the borderzone of the ischemic territory while only a few single such cells were found in the infarct core. All eGFP+ cells were co-labeled with the microglia/macrophage marker Iba-1 or the marker of reactive astrocytes, GFAP (Figure 5, top), but not with the mature neuronal marker NeuronalNuclei (NeuN) (data not shown). Interestingly, very proximal to the lesion area several GFAP^+^ cells were found, co-labeled with the eGFP antigen, while eGFP^+^/GFAP^+^ co-labeling was reduced in more distal regions. Imaging reporter presence in microglia was confirmed by RNA-ISH. Luc2 RNA molecules were found along with cells positive for Iba-1 RNA (Figure 5, bottom), especially close to the lesion core.

### Ym1 Up-Regulation After Stroke

**Figure 6:**
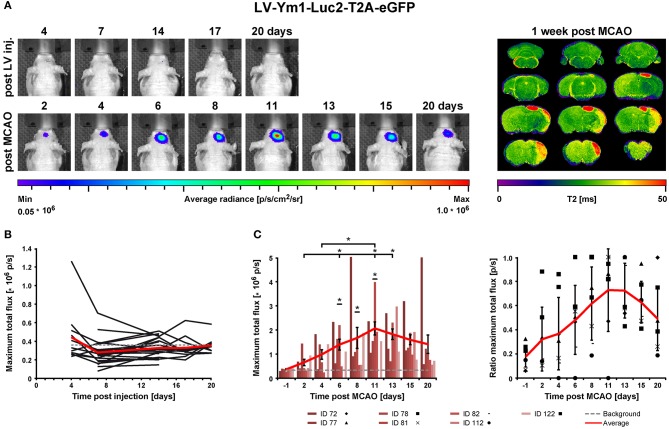
BLI *in vivo* analysis of Ym1 activity before and after stroke induction. **(A)** Longitudinal BLI signal development in one representative mouse before (upper row) and after MCAO (bottom row) with the corresponding MRI T2 map on the right. After stroke, signals increased strongly in animals with cortico-striatal lesions. **(B)** Before MCAO, BLI signal intensities, except for one animal, were below or close to the background threshold (gray dotted line). **(C)** After MCAO, BLI signal continuously increased in animals with cortico-striatal lesions until day 11, which was significantly different between 6 and 11 dps compared to all time points before stroke induction. BL signals on (6, 11, and 13) dps were significantly increased compared to 2 dps, and BLI signals on 11 dps were significantly increased compared to 4 dps. Raw data of maximum BLI signals are presented as total flux in the left diagram. Error bars represent ± SEM (left). In the right diagram, the values of the raw data were normalized to the maximal BLI signal of each individual, identified in the left diagram. Error bars represent ± STD (right). The red lines indicate average signals of each time point and the gray dotted line the background threshold. Wilcoxon Signed-Rank test was performed, *p ≤ 0.05. IR, imaging reporter (Luc2-T2A-eGFP); p/s, photons per second.

Before stroke, BLI signals were generally below or close to the signal background threshold except one outlier (Figures 6A,B), in line with reports of absent Ym1 expression in the healthy brain (28–30). After stroke, BLI signals strongly increased in animals with cortico-striatal lesions (Figure 6C) with a peak at 11 dps and an average 6-fold increase compared to the last days before stroke induction (i.e., 13 dpi or 14 dpi), followed by a slight decrease. BLI data were tested for statistical significance for all time points by the nonparametric Friedman test followed by the Wilcoxon Signed-Rank test. Photon emission after stroke was significantly increased for the time points 6–11 dps after stroke compared to all time points before stroke induction. Further, BLI signal intensities on 6 (*p* = 0.028), 11 (*p* = 0.028), and 13 dps (*p* = 0.043) were significantly higher compared to 2 dps, and BLI signal intensities on 11 dps were significantly increased relative to 4 dps (*p* = 0.028) (Figure 6C). Signals at the last time point (20 dps) were still higher than before stroke. The time points of maximum photon emission after stroke induction varied among the cortico-striatal lesioned animals: signals peaked at 6 dps (*n* = 1) and at (8, 11, or 13) dps (*n* = 2 for each time point). Additionally, in animals with cortico-striatal lesions, photon emission intensities differed inter-individually with maximum values ranging from 2.3-fold to 16.2-fold. In animals with T2 lesion volumes <5%/TB, BLI signals remained absent or constant at low levels (Supplementary Figure S4), thus confirming the ischemic damage as the induction for the observed up-regulated Ym1 activity. Contrary to the findings of the LV-iNOS-IR study, only weak or no linear positive correlations between BLI quantification (signal integration over time, maximal BLI signals, time to peak) and T2 lesion volumes at 7 dps were found.

**Figure 7:**
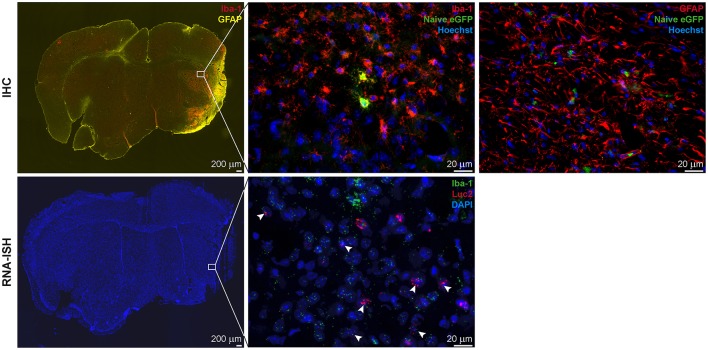
IHC and RNA-ISH of LV-Ym1-IR injected brains at 21 days after MCAO. IHC on top, RNA-ISH below. Iba-1 and GFAP antigens were strongly expressed in the ischemic area **(Left)**. Some eGFP^+^ cells co-expressed Iba-1 **(Center)**, but not GFAP **(Right)**. RNA-ISH confirmed reporter gene expression. Some Luc2 RNA positive cells co-expressed Iba-1 RNA. 4X magnifications left; 60X remaining images.

*Ex vivo* validations resulted in Iba-1^+^ microglia/macrophage accumulation in and around the lesion site and GFAP^+^ reactive astrocyte delineation of the lesion area. For the animals with cortico-striatal lesion, the presence of eGFP^+^ cells was confirmed by IHC inside the ischemic core as well as in the penumbra (Figure 7 top). eGFP^+^ cells co-labeled with Iba-1 but not with GFAP or NeuN. In these animals with cortico-striatal lesions, RNA-ISH confirmed the presence of Luc2 RNA positive cells within the same area that were also positive for Iba-1 RNA molecules (Figure 7 bottom). The two mice with T2 lesion volumes <5%/TB showed elevated T2 relaxation times only in the striatum, or in the striatum and only slightly in the cortex, which was in good accordance with Iba-1 and GFAP distribution (data not shown). In line with the BLI data, in the mice with T2 lesion volumes <5%/TB, only very few eGFP^+^ cells, also co-expressing Iba-1, were detected.

In summary, Ym1 promoter activity was strongly up-regulated after stroke and BLI signals remained elevated for at least up to 20 dps. Signal increases between 6 and 11 days post stroke were significantly different from signal intensity at all time points before stroke induction. In line with our hypothesis that focal ischemia causes Ym1 gene induction, BLI signal intensities did not increase in mice with T2 lesion volumes <5%/TB. No correlations were detected between BLI quantification and lesion size.

## Discussion

In the present investigation, we report the *in vivo* monitoring of the time profile of microglia polarization phenotypes after stroke. This study unravels for the first time the inter-individual variation in polarization intensity and speed (time-to-peak) of gene expression of pro-inflammatory and anti-inflammatory phenotypes. We have also for the first time analyzed the ischemic lesion in relation to the pro-inflammatory microglia phenotype, represented by iNOS expression, and found a correlation between the lesion size and the time-to-peak for the maximal iNOS expression. Such correlation was not observed for the anti-inflammatory Ym1 expression.

We have developed a protocol to assure a sensitive and stable signal of gene reporters and to permit safe assignment of all signal changes after stroke induction to microglia, separate from invading monocytes. The gene reporter signal of lentiviral constructs in healthy mouse brain shows that strong reporter expression is reached after ~2 weeks and reporter expression remains strong up to at least 5 weeks after lentiviral injection. Thus, stable gene expression condition is assured by the time of stroke induction, 2–3 weeks after virus injection. In consequence, signal changes after stroke induction can safely be assigned to up-regulation of the iNOS and Ym1 gene, respectively. Flow cytometry results demonstrate that the amount of monocytes, invaded in response to virus injection and therefore transduced to express the gene reporter, remains in the 1–2% range (cf. Figure 2) of the reporter signal contributed by microglia. It thus appears justified to speak of the signal as representing (almost) completely and exclusively microglia.

### iNOS

iNOS is a marker of the pro-inflammatory microglia phenotype (M1-like polarization) (4, 16, 19). The rather low iNOS signal in healthy brain increases ~3-fold within 3 days post stroke induction. The further development of the signal, continuously increasing or reversing already after a few days, however, varied among the individual animals. This inter-individual variation of the signal time profile prohibited conclusion of an average behavior pattern of iNOS expression over time.

Notably, imaging studies assessing the iNOS activity in real-time have yet to be done. Many studies focused on the very early acute time window up to 24 h after stroke onset. Studies assessing longer time intervals resulted in different temporal profiles of iNOS expression, maybe due to differing stroke models and/or strains of animals, but maybe also due to a cross-sectional approach smoothing out the rather heterogeneous iNOS responses. Wattananit et al. found significantly up-regulated iNOS mRNA levels only at 7 dps, but not on 3 dps and 14 dps (7) whereas the mRNA expression in the study by Hu et al. presented a continuous increase after stroke (4). Another study of permanent focal cerebral ischemia in mice reported a peak of ipsilateral iNOS mRNA levels at 96 h post stroke followed by a decrease at 7 dps (31). By FACS and qRT-PCR, iNOS was highly expressed in neutrophils and endothelial cells at 3 days post transient MCAO, which was attributed to a pathogenic effect on the post-ischemic brain (32). As we transduced cells prior to neutrophil infiltration, we exclude signal contamination from neutrophils. Likewise, morphologies on stained tissue ruled out signal origin from endothelial cells.

Many studies have assigned an important role to iNOS expression in stroke. But none of these investigations have related iNOS expression levels to lesion volumes. Strikingly, the major finding of our present iNOS study is an inverse correlation between the time-to-peak of iNOS expression and T2-weighted MRI lesion volumes on day 7 post stroke, indicating that larger lesions stimulate an earlier pro-inflammatory response. Further, it appears plausible that lesion severity and inflammatory reaction are mutually dependent and influence each other. Aswhal et al. found increased NOS activity in the lesion core compared to the penumbra up to 24 h post MCAO induction (33). Thus, increased NOS activity in the core could aggravate the lesion size. Further, Sehara et al. suggested that iNOS^+^ cells move from the lesion core into the peri-infarct region in the acute phase of stroke (34). Our analysis of BLI signal integration over time (total immune response) revealed a moderately positive correlation with T2 lesion volumes in our study. This result is in line with another report suggesting iNOS contribution to the extension of the lesioned tissue (31). Further, Vannucchi et al. reported that, 24 h after transient MCAO, numbers of activated iNOS^+^ microglial cells were high and nNOS levels significantly decreased in rats with worst motor performance, while rats with better motor performance lacked iNOS and expressed low levels of activated microglia, but showed consistent or increased numbers of nNOS^+^ neurons (35, 36).

### Ym1

Ym1 gene expression stands as a marker for the anti-inflammatory (M2-like polarization) microglia phenotype (4, 16, 19). In line with literature reports of missing Ym1 expression in healthy murine brain tissue (28–30), Ym1 BLI signals were missing before stroke. After stroke induction there was a strong, rather homogeneous rise in the BLI signal of the Ym1 gene reporter, rising out of the background noise level within 2 days. The Ym1 signal reached its maximum at 11–13 days post stroke induction, followed by a continuous decrease over the next week, but still remaining clearly above the noise level.

While several earlier studies agreed that Ym1 was mainly expressed during the first week, detailed analysis of the time point of maximal Ym1 expression varied substantially. Wattananit et al. found significantly increased Ym1 mRNA levels at 3 and 7 dps compared to the contralateral hemisphere without studying later time points (7). After permanent MCA occlusion, Perego et al. reported maximal Ym1 expression on IHC to occur already at 24 h with continuous decrease thereafter up to 7 dps (37). In their study, Ym1 expression was restricted to the lesion core and co-localized with CD206 or CD11b at both time points, and at 7 dps with the phagocytosis marker CD68. Interestingly, at 7 dps CD11b^+^/Ym1^+^ cells engulfed NeuN^+^ cells in the lesion core, indicating phagocytosis capacity, which was missing at 24 h. In all these studies, C57/Bl6 mice were used, which could explain discrepancies to our results on nude mice. Cuartero et al. found Ym1^+^ neutrophils at 24 or 48 h after permanent distal MCAO, which were phagocytosed by Iba-1^+^ microglia/macrophages. At this acute phase, they did not observe any Ym1^+^/Iba-1^+^ cell (38). As mentioned above, due to our study set-up, we rule out BLI signal contribution derived from neutrophils.

The protective action of Ym1 expression after stroke was shown in several studies. Fumagalli et al. (14) found increased Ym1 and decreased iNOS expression levels 24 h after transient MCAO in CX3CR1 deficient mice. These findings were associated with reduced infarct sizes (14). Most cells expressing Ym1, CD206, and iNOS co-labeled with CD11b^+^ cells. Transplantation of mesenchymal stem cells (MSCs) after transient common carotid artery occlusion resulted in up-regulated Ym1 expression, associated with an improved neurological outcome and reduced neuronal cell death (39).

In contrast to the M1-like response in our study, neither the time-to-peak, nor the signal integration over time (“total immune response”) correlated with T2 lesion volumes. Perego et al. found Ym1 expression in the lesion core only, whereas Yang et al. applying transient MCAO, observed Ym1 expression in active microglia in peri-infarct regions at 4 weeks post stroke (40). Hence, we suggest that the protective response is not directed to the lesion area itself but rather to the peri-infarct region. The M2-like phenotype microglia thus focuses more on salvaging still viable tissue, protecting it from further damage and eliminating dead cells and debris.

### Time Line of M1-Like or M2-Like Polarization

The present study of longitudinal monitoring of Ym1 (M2-like) or iNOS (M1-like) polarization phenotypes permits to determine the time profile on an individual basis. With this approach a clear maximum of Ym1 expression is found at 11–13 days, representing maximal anti-inflammatory microglia activity. Also, individually distinct time profiles were observed for iNOS expression, in clear dependence on the ischemic lesion severity.

Of course, this first long-term *in vivo* imaging trial is limited to only two marker genes. As is well known, antibody staining on nude mouse tissue is technically challenging, which is the reason why we could not directly validate our *in vivo* findings with other polarization markers on tissue samples *ex vivo*. It will be of great interest for future extensions of our present first step, to complement the present studies with those of imaging reporters under different polarization marker gene control.

Several earlier studies already focused on the M1- or M2-like polarization after stroke, but due to their restriction of single time point analysis in their invasive approaches, only selected time points after stroke were determined. There is general agreement that the M1-like phase follows the early M2-like polarization phase (4, 18–20). However, on detailed determination substantial disagreement is noted. By flow cytometry analysis on microglia Wattananit et al. revealed a significantly higher number of pro- (Ly6C^high^/CX3CR1^high^) than anti-inflammatory phenotype (Ly6C^low^/CX3CR1^high^) at 3 dps and 7 dps (7). Hu et al. found an early increase of iNOS mRNA levels, as well as of other pro-inflammatory markers, such as CD16, CD32, CD86, and CD11b from 3 dps on, with a continuous increase up to 14 dps, apart from CD86 (4). They further reported on maximal Ym1/2 mRNA levels on day 3 followed by a decline until 14 dps (4).

We propose that differences in temporal profiles compared to those of the literature are mainly based on the choice of stroke model, the use of different animal species or mouse strains, and, importantly, on the methodology. Many studies are based on qPCR, without considering cell specificity (4, 31, 41), so that distinction between monocytes, microglia and other cells was often not controlled. In our present study, the strategy of intrastriatal virus injections and later stroke induction assured signal emission from exclusively resident brain cells (with only 1–2% contamination from monocytes). Despite the invasiveness of intracranial injections, several reports argue for little microglia activation by lentiviral vector injection (42–44). To reduce the mechanical tissue damage to a minimum, ultra-thin capillaries with diameters of between 25 and 85 μm were used. With this approach, we kept microglia activation and infiltration of MDMs to a minimum, as confirmed by flow cytometry.

We want to emphasize here, that not only has the phenotypic diversity between different regions been observed for microglia in the healthy brain, but in addition, they react in a specific way after an insult react (45). Also, heterogeneity in the microglia transcriptome was reported between brain areas, resulting in three clusters separated by the cortical/striatal, hippocampal and cerebellar regions. Especially the cortical and cerebellar clusters predominantly revealed genes related to inflammatory response and regulation (46). Despite this heterogeneity, expression profiles of microglia residing in the striatum and cortex are similar with proliferation capacity in both regions after ischemia-induced tolerance (47, 48). Nonetheless, due to high specific microglia gene signature and function residing in different brain regions (47), we need to bear in mind that in our study we imaged microglia of the striatal region. Indeed, we had earlier already reported that microglia were mainly accumulated at the cortical infarct border, while in the striatal lesion territory, microglia were diffusely distributed across the whole ischemia area (49). Thus, it will be of great interest to extend our present study in future investigations to the behavior of microglia in cortex.

The generally invasive approaches of earlier studies had to rely on cross-sectional analyses, thus preventing the discrimination of inter-individual time profile variation. This also inhibited the here for the first time presented relation of iNOS expression to the lesion severity.

## Conclusion

Here we have focused on selective detection of one gene expression at a time, responsible for either anti-inflammatory or pro-inflammatory microglia phenotype. Present developments of quantitative detection of two different BLI gene reporters (50) will permit to monitor simultaneous anti- and pro-inflammatory microglia responses. We have shown a maximal M2-like, anti-inflammatory response at 11–13 days post stroke, independent of lesion severity, and an M1-like, pro-inflammatory response stimulated by the lesion severity. Taken together, the strategy presented here of noninvasive imaging for time profile monitoring helps to unravel the inflammatory response on an individual basis as a function of the disease state, e.g., lesion severity. Thus, this study has important implications for clinical translation. If confirmed by further studies, lesion severity may be considered a future surrogate marker corresponding to strength and speed of inflammation with the appropriate therapeutic approach.

## Ethics Statement

All animal experiments were approved by the local authorities [Landesamt für Natur, Umwelt und Verbraucherschutz North Rhine Westphalia (LANUV)] and conducted according to the German Animal Welfare Act under the animal permission: 84-02.04.2014.A226.

## Author Contributions

FC performed the experiments, analyzed the data, and wrote the manuscript. RP performed BLI experiments. SH-T and AM performed the stroke induction surgery. KF-D and CK performed the flow cytometry experiments. MA and MH designed the study and wrote the manuscript.

### Conflict of Interest Statement

MH is employed by Percuros B.V. The remaining authors declare that the research was conducted in the absence of any commercial or financial relationships that could be construed as a potential conflict of interest.

## Supplementary Material

Supplementary Figure S1 Flow cytometry gating strategy for analysis of LV-EF1α-IR injected brains at 15 dpi. Single cells were gated based on FSC-H and FSC-A (top left). From here, MNCs were gated based on SSC-A and FSC-A (top centre). MNCs negative for PI (top right) were analysed for CD11b and CD45 expression (centre, left) (CD11b^+^/CD45^low^ microglia, CD11b^+^/CD45^high^ MDMs) and for CD45 and eGFP expression (centre, right). CD11b^+^/CD45^+^ cells were analysed for GFP expression (bottom, left), and eGFP^+^ MNCs were analysed for CD11b and CD45 expression (bottom, right). The number of eGFP^+^ cells was low (left) and the majority of the cells were CD11b^−^/CD45^−^ (right). FSC-A, forward scatter area; FSC-H, forward scatter height; MDMs, monocyte derived macrophages; MNCs, mononuclear cells; PI, Propidium iodide; SSC-A, side scatter area.

Supplementary Figure S2 MRI Multislice T2 incidence maps at one week post stroke. Overlay of lesion sizes of LV-iNOS-IR (*n* = 5, left) and of LV-Ym1-IR injected mice (*n* = 13, right). The T2 maps were co-registered to a nude mouse brain template. Different colours symbolise the number of mice with lesions in the respective pixels. The maps of the LV-iNOS-IR and the LV-Ym1-IR animals include *n* = 3 and *n* = 9 mice with T2 lesion volumes >5%/TB and *n* = 2 and *n* = 4 with T2 lesion volumes < 5%/TB, respectively.

Supplementary Figure S3 BLI *in vivo* analysis of LV-iNOS-IR injected mice with small or absent ischaemic lesions. In animals with T2 lesion volumes < 5%/TB, BLI signals did not increase and were constant over time. **(A)** Representative images over time of one individual before (top) and after stroke (bottom). The corresponding T2 map is shown on the right. **(B)** For quantification, maximum photon emission was analysed. IR, imaging reporter (Luc2-T2A-eGFP); MCAO, middle cerebral artery occlusion; p/s, photons per second.

Supplementary Figure S4 BLI *in vivo* analysis of LV-Ym1-IR injected mice with small or absent ischaemic lesions. In mice with T2 lesion volumes < 5%/TB, no or very low BLI signals over time were observed. **(A)** Representative images over time in one animal with the corresponding T2 map on the right. **(B)** Signals were close to or below the background threshold (grey dotted line). The red line indicates the averaged BLI signals of *n* = 3 mice. Error bars represent + SEM.
