# Supplementary figures and images for: Individual in vivo Profiles of Microglia Polarization After Stroke, Represented by the Genes iNOS and Ym1
Source: Front Immunol. 2019 Jun 4;10:1236. doi: 10.3389/fimmu.2019.01236 (PMC6558167; doi:10.3389/fimmu.2019.01236)

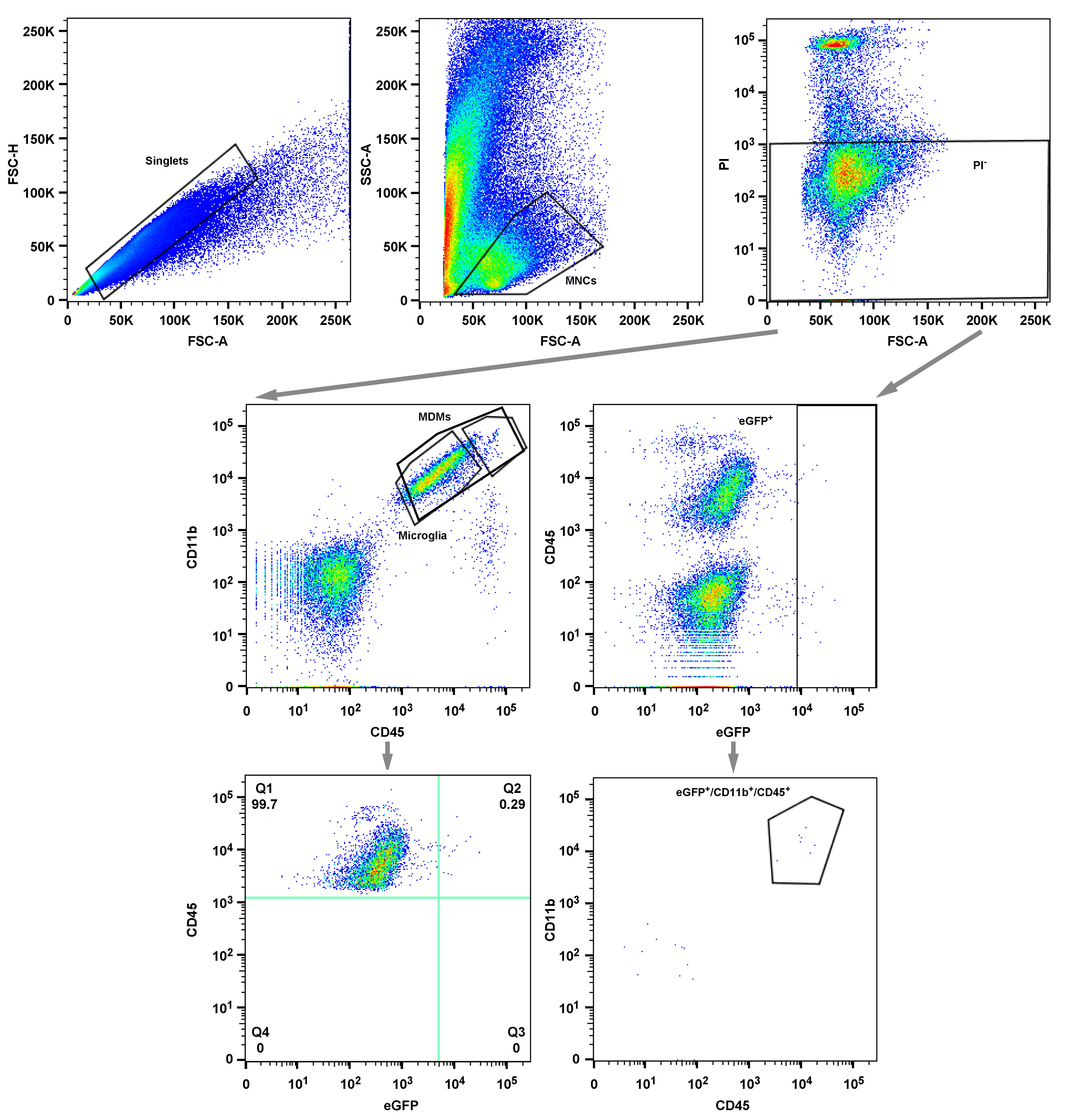

Supplement: Supplementary Figure S1 — Flow cytometry gating strategy for analysis of LV-EF1α-IR injected brains at 15 dpi. Single cells were gated based on FSC-H and FSC-A (top left). From here, MNCs were gated based on SSC-A and FSC-A (top centre). MNCs negative for PI (top right) were analysed for CD11b and CD45 expression (centre, left) (CD11b+/CD45low microglia, CD11b+/CD45high MDMs) and for CD45 and eGFP expression (centre, right). CD11b+/CD45+ cells were analysed for GFP expression (bottom, left), and eGFP+ MNCs were analysed for CD11b and CD45 expression (bottom, right). The number of eGFP+ cells was low (left) and the majority of the cells were CD11b−/CD45− (right). FSC-A, forward scatter area; FSC-H, forward scatter height; MDMs, monocyte derived macrophages; MNCs, mononuclear cells; PI, Propidium iodide; SSC-A, side scatter area. [file Image_1.TIF]

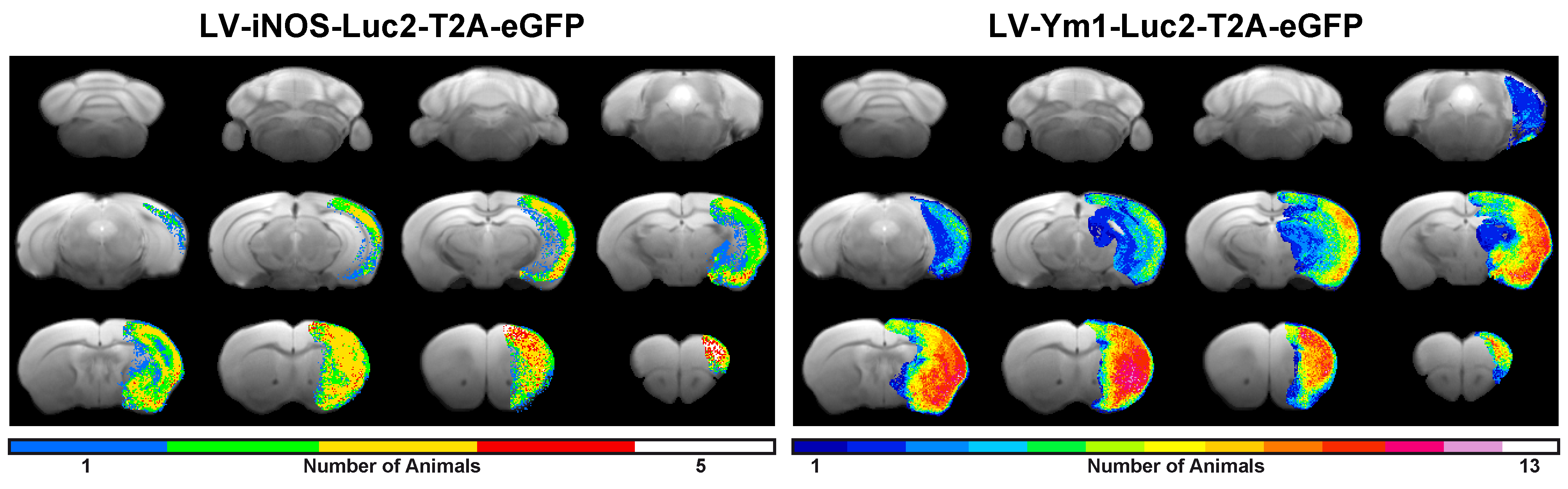

Supplement: Supplementary Figure S2 — MRI Multislice T2 incidence maps at one week post stroke. Overlay of lesion sizes of LV-iNOS-IR (n = 5, left) and of LV-Ym1-IR injected mice (n = 13, right). The T2 maps were co-registered to a nude mouse brain template. Different colours symbolise the number of mice with lesions in the respective pixels. The maps of the LV-iNOS-IR and the LV-Ym1-IR animals include n = 3 and n = 9 mice with T2 lesion volumes >5%/TB and n = 2 and n = 4 with T2 lesion volumes < 5%/TB, respectively. [file Image_2.TIF]

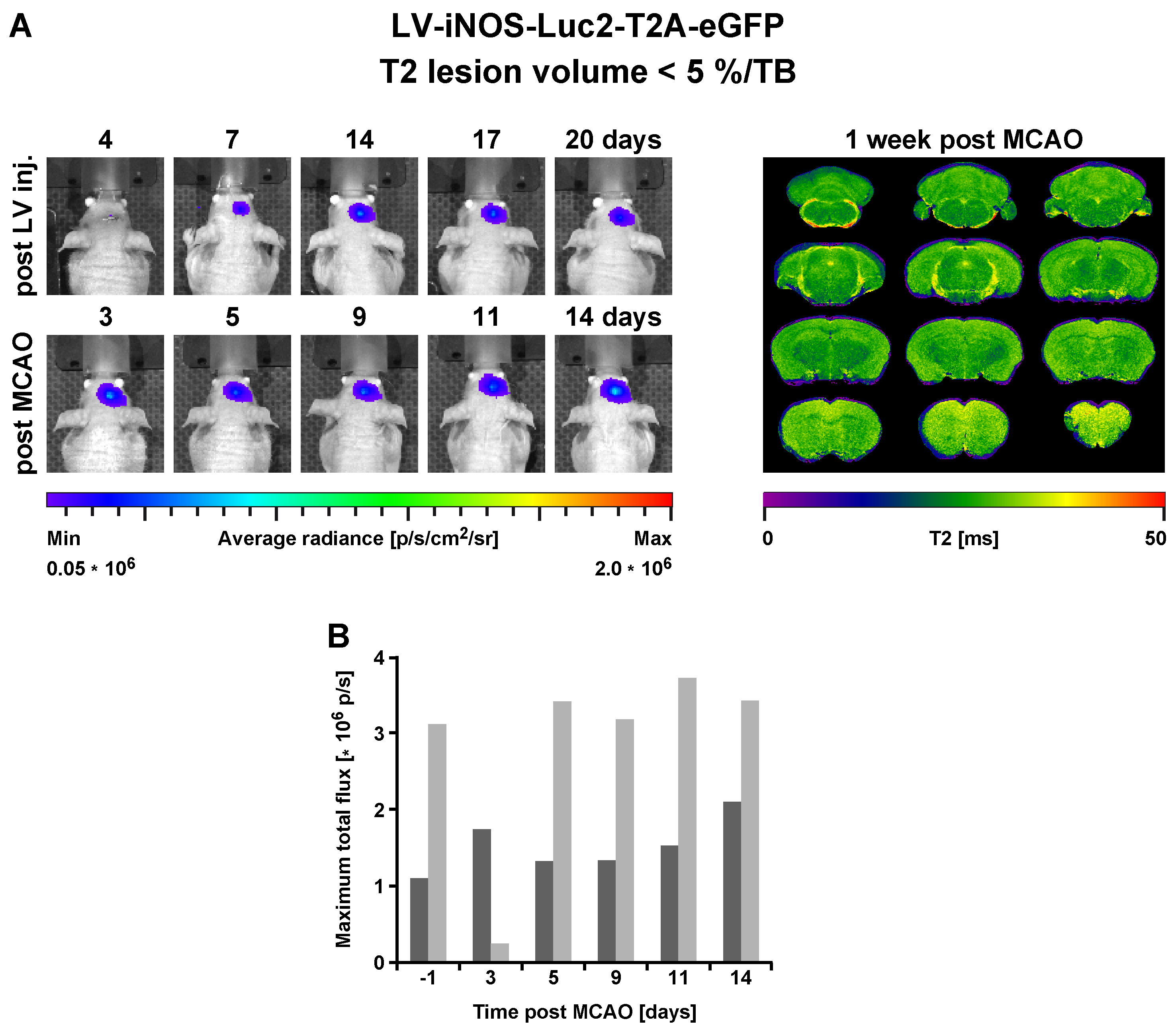

Supplement: Supplementary Figure S3 — BLI in vivo analysis of LV-iNOS-IR injected mice with small or absent ischaemic lesions. In animals with T2 lesion volumes < 5%/TB, BLI signals did not increase and were constant over time. (A) Representative images over time of one individual before (top) and after stroke (bottom). The corresponding T2 map is shown on the right. (B) For quantification, maximum photon emission was analysed. IR, imaging reporter (Luc2-T2A-eGFP); MCAO, middle cerebral artery occlusion; p/s, photons per second. [file Image_3.TIF]

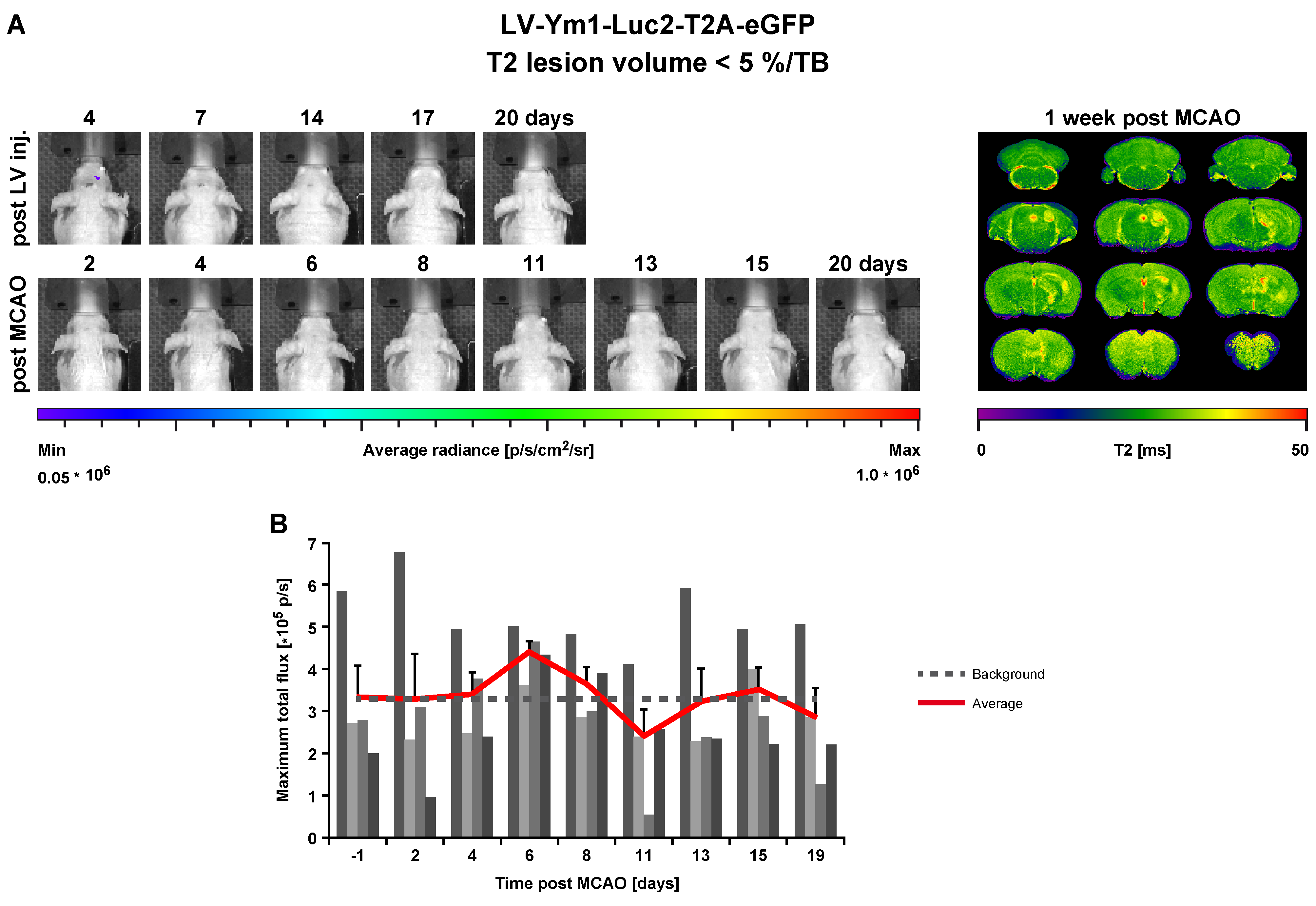

Supplement: Supplementary Figure S4 — BLI in vivo analysis of LV-Ym1-IR injected mice with small or absent ischaemic lesions. In mice with T2 lesion volumes < 5%/TB, no or very low BLI signals over time were observed. (A) Representative images over time in one animal with the corresponding T2 map on the right. (B) Signals were close to or below the background threshold (grey dotted line). The red line indicates the averaged BLI signals of n = 3 mice. Error bars represent + SEM. [file Image_4.TIF]
